# Unforeseen Complications: A Case of Subdural Anesthesia Post-epidural Insertion

**DOI:** 10.7759/cureus.45312

**Published:** 2023-09-15

**Authors:** Saleh A Ba-shammakh, Ahmad H Al-samnah, Marah W Qassim, Al-moutasm Bllah Z Al-shawabkeh, Qobol S Baamer

**Affiliations:** 1 Department of General Surgery, Princess Rahma Hospital, Irbid, JOR; 2 Department of Anesthesiology and Reanimation, The Islamic Hospital, Amman, JOR; 3 Department of General Surgery, Jordanian Royal Medical Services, Amman, JOR; 4 Department of Radiology, The Islamic Hospital, Amman, JOR

**Keywords:** subdural compartment, epidural anesthesia, cerebrospinal fluid, neuroepithelium, subarachnoid space, arachnoid mater, dura mater, meninges, neuraxial anesthesia, subdural anesthesia

## Abstract

Subdural anesthesia, although rare, is a significant complication of epidural anesthesia. This case report presents a 28-year-old female patient who developed sudden unconsciousness following epidural anesthesia administration for labor pain. Despite no evident contraindications to epidural anesthesia, she lost consciousness shortly after the initial test dose, leading to an emergency cesarean section under general anesthesia. The neonate showed signs of fetal bradycardia post-epidural and required intensive care. The patient made a complete recovery with no postpartum complications. This report underlines the need for vigilant monitoring and the importance of swift interventions in case of complications arising from epidural anesthesia.

## Introduction

Subdural anesthesia, which involves the administration of local anesthetic between the arachnoid and dura mater, is a significant yet often overlooked consequence of neuraxial anesthesia [1]. Historically perceived as a space filled with serous fluid, recent advanced microscopic investigations of post-mortem human samples indicate that this space might arise due to disease or medical intervention, instead of being a regular anatomical feature [2-3]. On a histological level, the so-called "subdural space" comprises a neuroepithelium characterized by unique elongated cells with sparse intercellular connections, encased by minimal collagen fibers and certain blood vessels, granting it low mechanical strength [4-5]. This space is sandwiched between the dura mater, which boasts roughly 80 layers of interconnected collagen, and the arachnoid mater, composed of multiple cell layers connected through specific desmosome-type connections [5]. The layout and features of the subdural neuroepithelium, combined with the external pressures it undergoes, dictate the clinical outcomes of substances delivered here. This distinctive interaction with local anesthetics can explain the broad range of clinical outcomes [6]. Clinically speaking, the nature of anesthesia hinges on which layers of the meninges are pierced. An intact dura mater reflects epidural anesthesia while penetrating just the dura mater but not the arachnoid results in subdural anesthesia. If the anesthetic accesses the subarachnoid space, it's recognized as spinal or subarachnoid anesthesia [7-8]. According to Lubenow's findings, the general population has an incidence of around 0.87% [6], and for pregnant women receiving epidural anesthesia, it's 0.024% [9]. Nonetheless, imaging studies suggest a range between 1% and an upper limit of 13% [10]. Due to its varied clinical symptoms, identifying subdural blocks is tricky. However, the recent introduction of methods like those from Lubenow et al. and Hoftman and Ferrante have furnished solid tools to improve diagnosis [1,6,11]. This introduction paves the way for in-depth discussions about atypical manifestations, connected dangers, and possible preventive steps regarding subdural anesthesia.

## Case presentation

A 28-year-old female, para 1 and gravida 2, presented with labor pain at 40 weeks and one day of gestation. A past obstetric event was documented involving a normal delivery with epidural insertion. No medical, surgical, or allergic histories were identified. Admission vitals included a BP of 126/81, heart rate (HR) of 82, respiratory rate (RR) of 20, oxygen saturation (SpO2) of 96%, and a temperature of 37°C. Clinical examination revealed a stable, alert, and oriented female in labor with no peripheral edema or signs of preeclampsia. Cardiovascular, respiratory, neurological, and abdominal assessments were normal, with a cervical dilation at 3 cm and 60% effacement. Considering her pain levels, an epidural block was initiated after ensuring no contraindications. The procedure was performed using a Tuohy needle, identifying the epidural space through the loss of resistance technique with normal saline and air.

**Figure 1:**
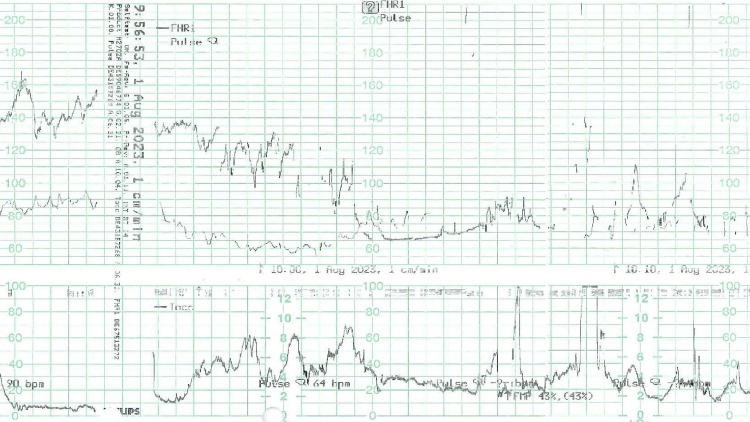
Cardiotocography (CTG) CTG following epidural insertion illustrated a notable deceleration in fetal heart rate, plummeting from a baseline of 130 bpm to a concerning 60 bpm, indicative of acute fetal bradycardia. This significant dip was captured during a critical phase post-epidural insertion.

Following a test dose of 3 cc lidocaine 2%, the loading dose was administered with 50 micrograms of fentanyl, 2 cc of bupivacaine 0.5%, and 5 cc of lidocaine 2%. Unexpectedly, the patient experienced dizziness leading to unconsciousness within 10 minutes. Her blood pressure declined from 110/70 to 95/55 mmHg, though the SpO2 remained stable at 95% on room air. Despite the clinical presentation, tests, including the glucose check for the aspirated fluid, showed no glucose, and the fluid was not warm to the touch on the skin, providing no evidence of a cerebrospinal fluid puncture, which excluded dural focus shifted to immediate management. The epidural infusion was halted, an oxygen mask was provided, and 500 cc of puncture and high spinal anesthesia was given. The operating room did not have immediate access to X-ray, MRI, or CT scans. Thus, the normal saline 0.9% IV bolus was administered. Concurrently, fetal cardiotocography (CTG) detected a concerning deceleration in fetal heart rate from 130 bpm to a mere 60 bpm, signaling acute fetal bradycardia post-epidural insertion (Figure 1).

The situation warranted an emergency cesarean section under general anesthesia using a rapid sequence induction, incorporating propofol 200mg, rocuronium 50mg, cefazolin 1g, paracetamol 1g, and 1 L of normal saline with 500 ml of RL. Oxytocin 40 IU and fentanyl 0.1 mg were administered once the baby was delivered. After delivery, the neonate presented an Apgar score of 7 and 8 at the 1-minute and 5-minute marks, respectively. Despite no improvement on the nasal cannula, the baby was admitted to the neonatal intensive care unit (NICU), requiring Vapotherm® at 5L/min. A subsequent X-ray revealed mild bilateral infiltration. Additionally, the baby was initiated on amikacin 63 mg Q24 and cefotaxime 200 mg Q8 IV. Lab tests for the neonate, including complete blood count (CBC), C-reactive protein (CRP), blood culture, prothrombin time (PT), partial thromboplastin time (PTT), international normalized ratio (INR), and kidney function test (KFT), returned normal results. The NICU stay totaled two days, with a discharge summary highlighting a healthy baby.

**Figure 2:**
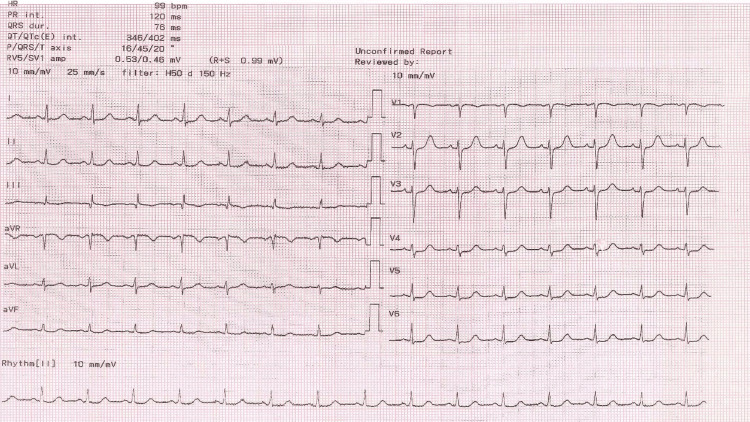
Electrocardiography (ECG) ECG showing a normal sinus rhythm with a heart rate of 99 beats per minute, indicating a regular rhythm. No abnormalities were detected in the waveforms, indicating normal cardiac electrical activity.

Post-cesarean, the mother regained consciousness upon extubation and remained alert and oriented during her recovery. An ECG performed on her showed normal results (Figure 2).

Initially, she displayed ankle flexion but was unable to flex the hip and knee. However, within 30 minutes of recovery, she regained full mobility in the knee and hip. An hour later, she was moved to the ward, demonstrating free movement across all limbs, spontaneous breathing, and an O2 saturation of 96% on room air. She was discharged the next day. Emotionally, she expressed frustration due to her baby's NICU admission but managed to breastfeed without complications. No postpartum complications were documented, or recommendations for future pregnancies were made.

## Discussion

The subdural compartment, which runs from the head down to the second sacral bone, carries specific anatomical attributes crucial for understanding subdural anesthesia [12]. It is usually more expansive in the neck region and becomes slender as it descends toward the lower back, resulting in varied manifestations of subdural blocks. This specific anatomy can lead to the preservation of certain sympathetic and motor capabilities during subdural administration. Namely, the dura and arachnoid mater have clear attachment points over the back part of the spinal nerve root, increasing space there. In contrast, the front part of the spinal nerve's coverings are more closely connected, limiting this space [11]. This space differential implies that subdural applications tend to accumulate posteriorly, leaving the anterior spinal nerves, mainly controlling sympathetic and motor operations, unaffected.

The effects and duration of a subdural block hinge on how the anesthetic disseminates. It typically starts slowly, taking hours, and the sensory blockage seems to exceed what one would expect for the given drug amount [13-14]. Contrary to the profound low blood pressure and lack of breathing seen in subarachnoid blocks, these are not usual for subdural blocks. Though challenging procedures, excessive needle adjustments, and previous spine surgeries can increase the likelihood of subdural blocks, new research indicates that even skilled doctors can accidentally induce them [15].

Diagnostic models, such as the one proposed by Lubenow et al., detail the criteria to determine the existence of a subdural block [1]. Primary signs comprise a negative cerebrospinal fluid test and an unusually extensive sensory block. Secondary indicators involve delayed sensory or motor blockages, inconsistent motor blockage, and disproportionate loss of sympathetic tone relative to the anesthetic dose [1]. Another method by Hoftman and Ferrante recommends steps incorporating dermatome evaluations, latency assessments, and other criteria like cardiovascular consistency, uneven distribution, and cranial nerve effects [6].

In subdural anesthesia administration, a needle typically pierces the dura mater, sometimes reaching the arachnoid membrane. The pressure difference between subdural tissue and cerebrospinal fluid becomes crucial in determining the direction of the anesthesia injection [3]. When the arachnoid remains untouched, the cerebrospinal fluid test would show a negative result, complicating the diagnosis [3].

Conditions like post-spinal tap complications, rotating the epidural needle, history of spinal surgeries, and multiple puncture attempts can also increase the odds of subdural blocks, irrespective of the doctor's expertise [16-17]. In a clinical context, outcomes can differ, ranging from typical epidural anesthesia to unconventional manifestations like one-sided or atypical blockages or even pronounced sensory blocks with minimal motor effects [8,18]. This diversity originates from the spaces and layers the injected solution accesses within the "subdural compartment" [4-5].

For a more definitive diagnosis, using contrast agents followed by imaging techniques, such as radiographs, fluoroscopy, or CT scans, can prove useful [1,15]. However, certain symptom markers, like a headache after application or a noticeable resistance loss, can signal a possible subdural block, prompting a shift in approach or imaging confirmation [16-17]. The test dose typically employed for epidural anesthesia might not be adequate to detect subdural blocks, but nerve stimulation techniques have shown potential in differentiating them [3].

Grasping the nature of subdural anesthesia mandates a deep understanding of the subdural compartment's structure, block occurrence mechanics, and the spectrum of clinical outcomes. Recognizing potential triggers, hazards, and foundational mechanics is pivotal for prevention, underscoring the importance of ongoing training and methodological refinement [16-17].

## Conclusions

Subdural anesthesia remains an elusive and unpredictable complication of epidural anesthesia. The presented case emphasizes the importance of being alert to sudden changes in maternal consciousness and immediate response to complications following epidural anesthesia. Although the patient recovered without any significant postpartum issues, the neonate faced transient distress. This report underscores the significance of early diagnosis, prompt intervention, and the continuous updating of anesthesia techniques and monitoring to ensure the safety of both the mother and the neonate.
